# Destructive interparental conflict affects Chinese children’s emotional and behavioral problems: Indirect pathways *via* parent–child attachment and emotional insecurity

**DOI:** 10.3389/fpsyg.2022.1024325

**Published:** 2022-11-16

**Authors:** Meirong Yang, Huan Qi, Zhaoyan Meng, Xiangfei Duan, Libin Zhang

**Affiliations:** ^1^School of Psychology and Mental Health, North China University of Science and Technology, Tangshan, Hebei, China; ^2^School of Educational Science, Ludong University, Yantai, Shandong, China; ^3^Collaborative Innovation Center of Assessment Toward Basic EducationQuality, Beijing Normal University, Beijing, China

**Keywords:** destructive interparental conflict, parent–child attachment, emotional insecurity, Chinese children, emotional and behavioral problems

## Abstract

**Background:**

Previous studies have demonstrated that destructive interparental conflict (IPC) is closely related to the emergence of emotional and behavioral problems in adolescents. In addition, in the family system, such conflict also affects the patent–child attachment relationship and emotional insecurity of adolescents.

**Objectives:**

This study mainly explores the relationship between destructive interparental conflict and adolescents’ emotional and behavioral problems, focuses on the role of parent–child attachment and emotional insecurity, and analyzes whether this relationality plays multiple mediating roles in the influence of destructive interparental conflict on emotional and behavioral problems.

**Methods:**

Data for the study were obtained through a questionnaire survey conducted on 524 Chinese adolescents from primary and junior high school.

**Results:**

Structural equation modeling was conducted to test direct and indirect pathways between destructive interparental conflict and Chinese adolescents’ emotional and behavioral problems. Destructive IPC negatively predicted parent–child attachment and parent–child attachment negatively predicted emotional and behavioral problems. Destructive Interparental conflict positively predicted emotional insecurity and emotional insecurity positively predicted emotional and behavioral problems.

**Discussion:**

The results show that: (1) Parent–child attachment negatively predicted emotional and behavioral problems, and emotional insecurity positively predicted the same. (2) Parent–child attachment and emotional insecurity act in a multiple mediating role between destructive IPC and adolescents’ emotional and behavioral problems. (3) Parent–child attachment and emotional insecurity constitute two indirect pathways between destructive IPC and adolescents’ emotional and behavioral problems, respectively.

**Conclusion:**

Destructive IPC can adversely affect emotional and behavioral problems among adolescents; destructive IPC plays a damaging role in their emotional security and parent–child attachment, consequently effecting emotional and behavioral problems.

## Introduction

### Adolescents’ emotional and behavioral problems

Early adolescence is a critical developmental period for emotional and behavioral problems (Yang et al., 2019), with the emotional and behavioral problems that commonly emerge during this time ascending the risk of life-long impairment (Patton et al., 2016). Adolescents with such emotional and behavioral problems often tend to be asocial and show poor peer interaction, emotional instability (Weymouth et al., 2019), depression, and anxiety (Ran et al., 2021). There is evidence that children with moderate to severe emotional and behavioral problems in the preschool period are difficult to change in the adolescents and develop dysfunction, negatively impacting their peer relationships, self-development, academic, and family function over the long-term (Liu et al., 2022). Whether it is for individuals, families, or society as a whole, adolescents’ emotional and behavioral problems should attract sufficient attention. In this regard, then, studying the influence factors of adolescents’ emotional and behavioral problems is significant for the development of intervention measures that reduce such behavior and improve adolescents’ mental health.

### Destructive interparental conflict and adolescents’ emotional and behavioral problems

Destructive interparental conflict refers to verbal or physical attacks or disputes between husband and wife, due to disagreement or other reasons (Chi, 2003). Moreover, the parental relationship has a general influence on adolescents’ self-awareness, personality characteristics, mental health, and behavior (Lucas-Thompson et al., 2015). Research shows that children from divorced but conflict-free homes have fewer emotional and behavioral problem than children whose parents remain in an unhappy marriage (Borrine et al., 1991), adolescents who grow up in a family with a harmonious parental relationship and warm atmosphere have a higher level of self-awareness than those who grow up in families with a tense parental relationship; the latter tend to become confident and full of happiness and hope for the future.

The quality of the parental relationship is considered a key factor in the psychopathology of adolescents and adolescents (Harold and Sellers, 2018). In the early development of infants, parents’ neglect and abuse affect adolescents’ neurodevelopment (Davies et al., 2017). Emotional neglect of adolescents, caused by destructive Interparental Conflict (IPC), will continue to affect the emergence of adolescents’ emotional and behavioral problems, especially leading to depression and low ability in emotional adjustment and social adaptation (Davies et al., 2017), Most such problems are connected to growing up in disharmonious and unhappy families. Compared with those who have experienced constructive IPC, adolescents with destructive IPC will have more emotional and behavioral problems (Adare et al., 2021). Since witnessing destructive IPC, or experiencing parents’ divorce, is the primary adverse event in adolescents’ growth period, it is closely related to their emotional and behavioral problems. Therefore, this study intends to investigate the relationship and possible pathways between destructive IPC and adolescents’ emotional and behavioral problems among Chinese adolescents.

### An indirect pathway through child–parent attachment

Based on *Attachment Theory*, parents form the source for adolescents’ initial emotions (Tracy, 2001). Parent–child attachment formed under a harmonious parent–child relationship can promote adolescents’ healthy emotional development. Attachment is a primary side of the parent–child relationship that makes adolescents feel secure and unthreatened in the family environment (Ali et al., 2021). Research revealed that insecure attachment was negatively correlated with both social adjustment and mental health (Brumariu and Kerns, 2010). In the view of life history (LH) theory, the attachment styles in childhood interact with other behavioral developments (Hawley et al., 2009), at the same time, fast LH strategies, i.e., less investment in adolescents’ growth and less attention to adolescents’ learning, cognitive development, and parenting are often associated with unsafe and unpredictable adolescents’ environments, negative environmental influences can be up-regulated or exacerbated with insecure attachment (Lu and Chang, 2019; Lu et al., 2022). Unpredictability experienced by children before the age of 10, such as family dislocation, unstable family relationships, positively predicts aggressive behaviors in adolescent and young adulthood (Ellis et al., 2021). Children in insecure attachment families will tend to adopt coercive strategies to compete with their peers in the face of adverse social realities (Chen and Chang, 2012). In the family environment parents’ marital relationship and quality will also affect the parent–child relationship, and the level and quality of parent–child attachment to a certain extent. Therefore, parents’ neglect of adolescents’ emotions caused by destructive IPC has indirect and inverse effects in predicting safe child–father attachment (Schudlich et al., 2019).

Parents’ participation reduces adolescents’ anxiety and attachment (Barone et al., 2021). Attachment plays an important role in the development of adolescents’ emotional skills. Adolescents in secure attachment recognize emotional expressions of others faster than in insecure attachment (Mirbagheri et al., 2020).

A good parent–child attachment relationship can positively predict behaviors and habits among Chinese adolescents (Mo et al., 2021). Frequent parental conflicts can cause parents to ignore adolescents’ feelings and tend to invest little and do not interact with adolescents. This can lead to lower levels of parent–child attachment, which, in turn, can increase the risk of adolescents’ emotional and behavioral problems. Therefore, in this study, we regard parent–child attachment as one of the important predictors of adolescents’ emotional and behavioral problems.

### An indirect pathway through emotional insecurity

Emotional security is a sense of security, stability, and happiness that adolescents obtain in a safe family environment and stable parental relationship. Emotional Security Theory (EST; Davies et al., 2016) explains that a high degree of emotional insecurity among adolescents is caused by their involvement in frequent and intense exposure to destructive IPC. This mainly includes two aspects: negative emotional response, stress behavior response. Existing studies have indicated that the adjustment of husband and wife is an important factor in forming the family emotional atmosphere, which helps to increase resiliency in the face of stressful life events, and is of great significance to family health. Therefore, when negative interparental behaviors, which also comprise destructive IPC, are buffered by positive interactions, they will pose a lesser threat to the child’s sense of security (Zemp et al., 2019). Furthermore, when a child feels that the stable family structure is threatened under destructive IPC, they become emotionally disturbed, and even use a negative or avoidant posture to protect themselves, connecting with school problems (Martin et al., 2017). In short, emotional insecurity is a lack of security caused by unexpected life events during childhood.

Furthermore, such adolescents with emotional insecurity also show poor sleep quality, which, in turn, leads to poor academic performance and poor interaction with friends; the latter also leads to poor social skills (Davies et al., 2017), bad peer relationship, and other behaviors problem, for instance, internet addiction (Zhou et al., 2017). Previous studies have concluded that the quality of the mother’s emotion and emotional attachment relationship will be carried over to the child across generations (Cooke et al., 2019). When the mother is in conflict with her spouse, adolescents will also experience similar negative emotional reactions in the family environment, and this can lead to a decreasing quality of the attachment relationship. Furthermore, this insecurity and emotional and behavioral problems will continue to remain for some time (Carlone and Milan, 2021). Insecurities in emotional experience are reinforced by frequent family conflicts. Studies have also found that emotional insecurity can predict the relationship between parents’ marital conflict and adolescents’ emotional and behavioral problems (Davies et al., 2017), Therefore, we regard emotional insecurity as one of the important predictors of emotional and behavioral problems among Chinese adolescents.

Destructive IPC will have immediate and long-term negative effects on adolescents’ emotional and behavioral problems. Its immediate influence will make adolescents exhibit low and nervous psychological reactions when faced with conflict. Its long-term influence refers to a series of emotional and behavioral problems, such as unstable peer interaction, caused by destructive IPC, which eventually influences adolescents’ social interaction mechanism.

**Figure 1:**
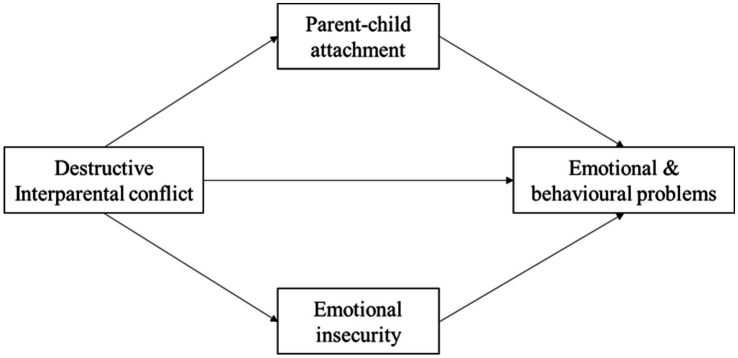
Hypothetical indirect pathways between destructive interparental conflict and adolescents’ emotional and behavioural problems.

In summary, based on the aforementioned theories and previous research, this study aimed to investigate the impact of destructive IPC on emotional and behavioral problems among Chinese adolescents and their internal mechanism, and proposed four hypotheses (as shown in Figure 1). (1) Destructive IPC is positively correlated with emotional and behavioral problems among Chinese adolescents; (2) Parent–child attachment can negatively predict emotional and behavioral problems and emotional insecurity can positively predict emotional and behavioral problems; (3) Parent–child attachment and emotional insecurity play an intermediary role between destructive IPC and Chinese adolescents’ emotional and behavioral problems respectively; and (4) Parent–child attachment and emotional insecurity play multiple intermediary roles between destructive IPC and adolescents’ emotional and behavioral problems.

## Materials and methods

### Participants and procedure

This study used convenient sampling, 524 participants from primary school and junior high school volunteered to participate in our research, 252 girls (accounting for 48.1%), 272 boys (accounting for 51.9%), *M_age_* = 13.69 years, SD = 1.37 years (min = 12, max = 17). The data were collected with the prior consent of the school teacher, and obtained the consent of the parents of the participants through the head teacher of the participants in 2018. Communicated with the head teacher to explain the purpose and requirements of this research, the head teacher and graduate students majoring in psychology were the testers, the questionnaire was distributed by class. Schools, parents, and adolescents are actively involved in data collection. In terms of the family structure, 467 participants were reported from nuclear family, 38 from single-parent family, and 19 from stepfamily. This study was approved by the Ethics Committee of North China University of Science and Technology.

Before the questionnaire completion, trained research assistants introduced the purpose of data collection, and provided guidance on how to fill out the survey. Participants were ensured that their responses will be anonymous and confidential.

### Demographic variables

Participants reported their sex (1 = boy, 0 = girl), age, family structure (1 = nuclear family, 2 = single-parent family, and 3 = stepfamily), and subjective socio-economic status (SES). Subjective SES was assessed by self-reported.

## Measures

### Children’s perception of interparental conflict scale

The Children’s Perception of Interparental Conflict Scale (CPIC) compiled by Grych et al. (1992) and revised by (Chi and Xin, 2003) was selected. The scale has ideal psychometric indexes and suitable for Chinese participants (Chi and Xin, 2003). The scale has 40 items, which are divided into two factors: conflict characteristics and conflict evaluation. The conflict characteristics include three conflict dimensions of frequency, intensity, and resolution, such as ‘My parents rarely quarrel.’ Participants rated each item on a 4-point Likert-type scale from 1 (*absolutely true*) to 4 (*not at all true*). This study only uses conflict characteristic factors to measure parental marital conflict. Scores were averaged across items, so that higher scores indicate the higher frequencies of conflict, higher intensity of conflict, and lower levels of resolution. Latent variable of destructive interparental conflict was constructed using the dimension scores of frequency, intensity, and resolution. The Cronbach’α coefficient of the scale was 0.910.

### The inventory of parent and peer attachment

The Inventory of Parent and Peer Attachment (IPPA Scale; Armsden and Greenberg, 1987) was selected, the scale has ideal psychometric indexes and suitable for Chinese participants (Ju et al., 2011), which was divided into two subscales: child–parent attachment and peer attachment. Each subscale groups into three dimensions: trust, communication, and alienation. As the research direction of this paper is the mediating role of parent–child attachment, we choose the subscale of parent–child attachment, which consists of 28 questions, including 10 questions of trust, 10 questions of communication, and 8 questions of alienation, adapting 5-point Likert’s scoring method, with scores ranging from 1 to 5. The response format was 1 (*never*) to 5 (*always*). Latent variable of parent–child attachment was constructed using the dimension scores of trust, communication, and alienation. The higher the latent variable score of participants, the better the attachment quality. The Cronbach’ *α* coefficient of the scale was 0.826.

### Security in the interparental subsystem scale

Security in the Interparental Subsystem Scale (SIS Scale; Davies et al., 2002) was used to assess how adolescents balance emotional security when facing the interparental conflict between parents in the natural environment. The scale consists of 37 items, including emotional reactivity, behavioral disorders, avoidance, involvement, constructive family representations, destructive family representations, conflict spillover representations, etc. Due to the lack of factor validity of the three factors of behavioral disorder, avoidance, and involvement in the original scale, then only three factors of emotional reactivity, destructive family representations, and conflict spillover representations were used as SIS scales for Chinese adolescents groups to measure the emotional insecurity of adolescents facing interparental conflict, which is revised by Wang et al. (2014). Latent variable of emotional insecurity was constructed using three factors in this study. The scale has ideal psychometric indexes and suitable for Chinese participants. The revised questionnaire combines destructive family representations and conflict spillover representations into the dimension of negative representation, which reflects the destructiveness of interparental conflict perceived by children to their own family happiness, and possesses good reliability and validity. The revised questionnaire consists of 17 questions (the dimension of emotional reactivity includes 9 questions, the dimension of destructive family representations includes 4 questions, and the dimension of conflict spillover representations includes 4 questions), adapting Likert’s 4-point scoring method. The response format was 1 (*not at all true*) to 4 (*absolutely true*). The Cronbach’ *α* coefficient of the scale was 0.922.

### Strengths and difficulties questionnaire

Difficulty and Strengths Questionnaire (SDQ Questionnaire) was adopted by Goodman (1997). The questionnaire was divided into parent version, teacher version, and student version, which was suitable for children and adolescents aged 4–16 years. In the study, the student version of SDQ was used for measurement, which is the simplified Chinese version published by SDQ official website.^1^ The research shows that the Chinese version of SDQ possesses good reliability and validity, and can be used to evaluate the mental health status of Chinese children and adolescents (Xu et al., 2019). Student version of SDQ questionnaire has 25 items, which are divided into five dimensions: conduct problems, emotional symptoms, hyperactivity-inattention, peer problems, and prosocial behaviors. Among them, 10 items are advantages, 14 items are difficulties, and 1 item is neutral. Each item is graded according to level 3, with 0 score for ‘*not at all true,*’ 1 score for ‘*somewhat true*’ and 2 points for ‘*absolutely true.*’ Among them, the 7th, 11th, 14th, 21st, and 25th items are entitled reverse scoring items, with 2 points for ‘*not at all true*’ 1 point for ‘*somewhat true*’ and 0 point for ‘*absolutely true.*’ We use these five dimensions to construct the latent variable to evaluate adolescents’ emotional and behavioral problems. The Cronbach’α coefficient of the scale was 0.710.

### Analytical procedure

First, Harman’s single-factor test was used to check for common method variance. Then, SPSS 25.0 was used to conduct descriptive statistics and zero-order correlation analyses controlling for age, gender, SES, and family structure. Finally, basing on latent variables constructing by the dimensions of each scale, the structural equation model was constructed by AMOS 24.0, with destructive IPC as the independent variable, adolescents’ emotional and behavioral problems as the dependent variable, and parent–child attachment and emotional insecurity as mediating variables. The model was assessed using a combination of indices and criteria, such as ×2/*df* < 5, CFI > 0.90, TLI > 0.90, RMSEA < 0.08, and SRMR < 0.05. The mediating effect was measured by bootstrapping with 5,000 resamples drawn to derive the 95% confidence intervals (CIs).

## Results

### Common method biases

Since interparental conflict, parent–child attachment, emotional insecurity, and emotional and behavioral problems are measured by self-report, there may be common method bias in this study, Harman’s single-factor test was used to verify common method bias (Podsakoff et al., 2003). Exploratory factor analysis was conducted on all variables, and the results of factor analysis extracted 20 factors with characteristic roots greater than 1. The first factor explained 22.21% of the total variation, lower than the critical point of 40%. Therefore, it was considered that there was no obvious common method bias in this study.

### Descriptive statistics of every variable

Means, SDs, and zero-order correlations among variables are shown in Table 1. The results showed that the dimensions of inappropriate resolution, frequency of destructive IPC, and intensity of destructive IPC are significantly negatively correlated with the dimensions of communication, trust, and alienation of parent–child attachment (*r* = −0.55 ~ −0.41, *ps* < 0.01), indicating that the higher the score of destructive IPC, the lower the score of parent–child attachment, the more serious destructive IPC and the worse the parent–child attachment. The dimensions of inappropriate resolution, frequency of destructive IPC, and intensity of destructive IPC are positively correlated with the dimensions of conflict spillover representations, destructive family representations, and emotional reactivity of emotional insecurity, respectively (*r* = 0.21 ~ 0.37, *ps* < 0.01), at the same time, the three dimensions of destructive IPC are positively correlated with the dimensions of hyperactivity-inattention, conduct problems, prosocial behaviors, emotional symptom, and peer problems of emotional and behavioral problems (*r* = 0.09 ~ 0.33, *ps* < 0.05), indicating that the higher the score of destructive IPC, the more serious the emotional insecurity and emotional and behavioral problems of Chinese adolescents. The dimensions of communication, trust, and alienation of parent–child attachment are negatively correlated with the dimensions of hyperactivity-inattention, conduct problems, prosocial behaviors, emotional symptom, and peer problems of emotional and behavioral problems (*r* = −0.51 ~ −0.20, *ps* < 0.01), indicating that the better the parent–child attachment, the less the adolescents’ emotional and behavioral problems. Apart from prosocial behaviors, the dimensions of conflict spillover representations, destructive family representations, and emotional reactivity of emotional insecurity are significantly positively correlated with hyperactivity-inattention, conduct problems, prosocial behaviors, emotional symptom, and peer problems of emotional and behavioral problems, respectively, (*r* = 0.14 ~ 0.45, *ps* < 0.01), indicating that the higher the emotional insecurity of Chinese adolescents, the more serious their emotional and behavioral problems.

**Table 1:** Zero-order correlations and descriptive for study variables.

	1	2	3	4	5	6	7	8	9	10	11	12	13	14	15	16
1 Gender (0 = girls, 1 = boys)	–															
2 Age	0.05	–														
3 Inappropriate resolution	−0.03	0.12^**^	–													
4 Frequency of destructive IPC	−0.07	0.10^*^	0.67^**^	–												
5 Intensity of destructive IPC	0.03	0.12^**^	0.66^**^	0.74^**^	–											
6 Communication	−0.02	−0.20^**^	−0.49^**^	−0.50^**^	−0.49^**^	–										
7 Trust	−0.03	−0.18^**^	−0.55^**^	−0.54^**^	−0.55^**^	0.81^**^	–									
8 Alienation	−0.01	−0.18^**^	−0.42^**^	−0.43^**^	−0.41^**^	0.60^**^	0.66^**^	–								
9 Conflict spillover representations	0.00	−0.07	0.272^**^	0.33^**^	0.29^**^	−0.22^**^	−0.33^**^	−0.45^**^	–							
10 Destructive family representations	−0.05	−0.16^**^	0.35^**^	0.37^**^	0.34^**^	−0.18^**^	−0.23^**^	−0.34^**^	0.61^**^	–						
11 Emotional reactivity	−0.11^*^	−0.08	0.21^**^	0.27^**^	0.26^**^	−0.10^*^	−0.14^**^	−0.30^**^	0.70^**^	0.70^**^	–					
12 Hyperactivity-inattention	0.11^*^	0.12^**^	0.32^**^	0.33^**^	0.31^**^	−0.42^**^	−0.47^**^	−0.42^**^	0.31^*^	0.22^**^	0.25^**^	–				
13 Conduct problems	0.09^*^	−0.04	0.26^**^	0.27^**^	0.26^**^	−0.33^**^	−0.40^**^	−0.44^**^	0.31^**^	0.16^**^	0.19^**^	0.45^**^	–			
14 Prosocial behaviors	0.18^**^	0.01	0.23^**^	0.23^**^	0.27^**^	−0.36^**^	−0.31^**^	−0.22^**^	0.00	0.05	−0.03	0.20^**^	0.17^**^	–		
15 Emotional symptom	−0.17^**^	0.08	0.26^**^	0.35^**^	0.26^**^	−0.38^**^	−0.39^**^	−0.51^**^	0.46^**^	0.38^**^	0.43^**^	0.43^**^	0.45^**^	0.08	–	
16 Peer problems	0.07	−0.03	0.10^*^	0.10^*^	0.09^*^	−0.24^**^	−0.21^**^	−0.25^**^	0.16^**^	0.14^**^	0.19^**^	0.24^**^	0.26^**^	0.14^**^	0.30^**^	–
*M*	–	–	10.50	13.06	14.87	28.09	35.28	17.34	7.03	7.60	17.79	3.55	2.41	2.59	3.08	2.81
SD	–	–	4.09	4.07	4.61	8.98	9.05	4.72	2.77	3.44	6.86	2.31	1.62	2.23	2.52	1.61

*M* = mean; SD = Standard deviation. ^*^p < 0.05; ^**^p < 0.01.

### Direct way and indirect way between destructive IPC and emotional and behavioral problems

**Figure 2:**
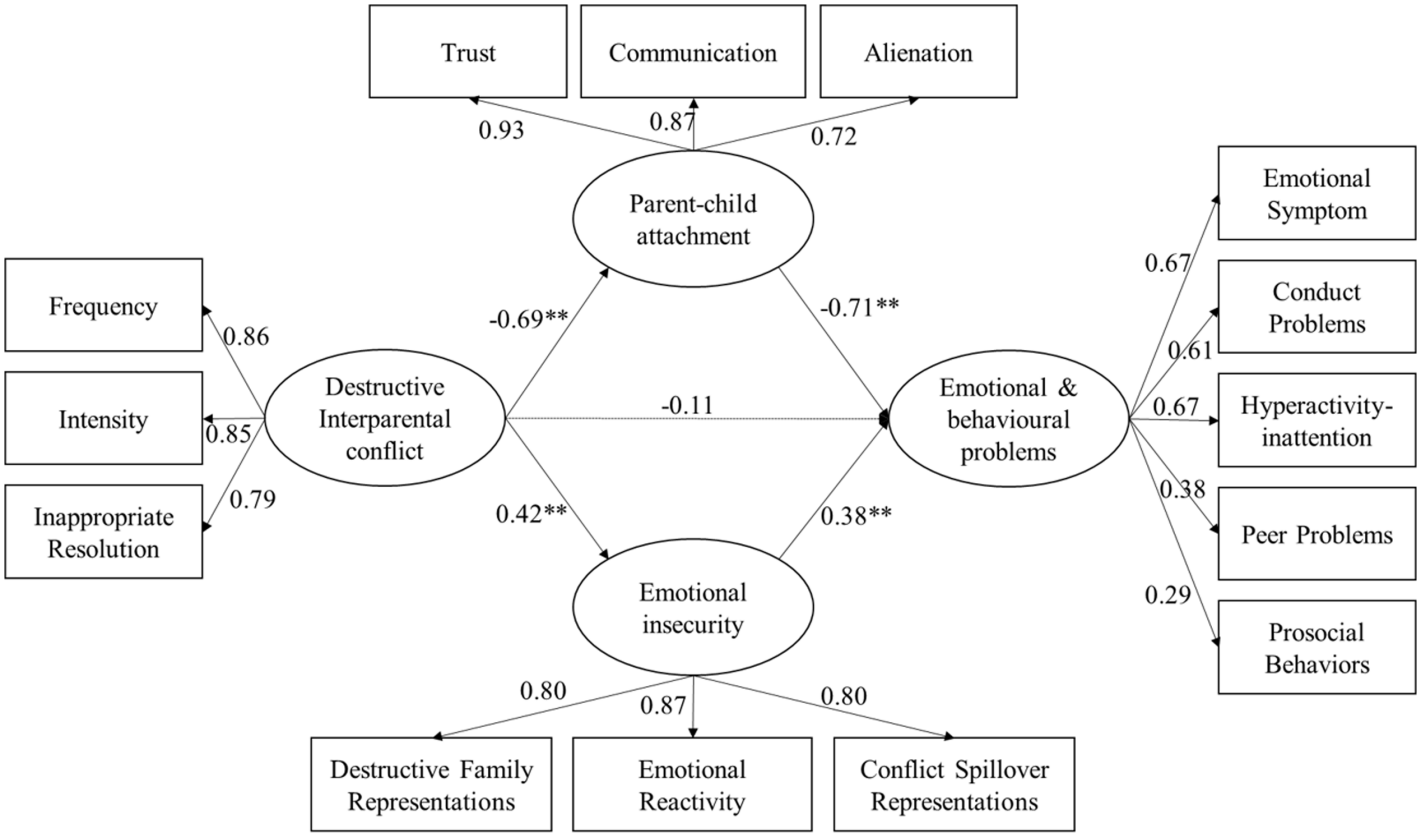
Estimates from SEM testing the direct and indirect pathways between destructive IPC and Emotional and behavioural problems. Estimates are standardized coefficients. The dashed line is the nonsignificant path. *p < 0.05; **p < 0.01. SEM = structural equation modeling.

Because destructive IPC, parent–child attachment, emotional insecurity, and emotional and behavioral problems are multidimensional, latent variable analysis is used in this study. As presented in Figure 2, SEM was conducted to test direct and indirect pathways between destructive IPC and Chinese adolescents’ emotional and behavioral problems. The model yielded good fit to the data, *χ*^2^/*df* = 3.35，RMSEA = 0.07, SRMR = 0.05, CFI = 0.93, TLI = 0.90. Destructive IPC negatively predicted parent–child attachment (*β* = −0.69, *p* < 0.01), and parent–child attachment negatively predicted emotional and behavioral problems (*β* = −0.71, *p* < 0.01). Destructive IPC positively predicted emotional insecurity (*β* = 0.42, *p* < 0.01), and emotional insecurity positively predicted emotional and behavioral problems (*β* = 0.38, *p* < 0.01).

### Bootstrapping analyses: Further testing the mediating roles of parent–child attachment and emotional insecurity

The mediating effect was measured by using the deviation correction percentile Bootstrap (repeated sampling 5,000 times). As represented in Table 2, the indirect effect of destructive IPC on emotional and behavioral problems through parent–child attachment is significant (*β* = 0.49, *p* < 0.01, 95%CI = [0.39,0.61]), and the indirect effect of destructive IPC on emotional and behavioral problems through emotional insecurity is significant (*β* = 0.16, *p* < 0.01, 95% CI = [0.10, 0.24]), indicating that parent–child attachment and emotional insecurity mediate the relation of destructive IPC and emotional and behavioral problems, respectively.

**Table 2:** Standardized direct and indirect paths between IPC and child’s emotional and behavioral problems.

Paths	*β*	SE	*p*	LLCI	ULCI
Total effect	0.539	0.055	<0.001	0.422	0.639
Direct effect					
IPC → emotional and behavioral problems	−0.111	0.091	0.208	−0.297	0.060
Total indirect effect	0.650	0.064	<0.001	0.532	0.785
IPC → parent–child attachment → emotional and behavioral problems	0.490	0.055	<0.001	0.392	0.611
IPC → emotional insecurity → emotional and behavioral problems	0.160	0.038	<0.001	0.096	0.244

## Discussion

This study investigated the relationship between destructive IPC and emotional and behavioral problems among Chinese adolescents, the pathways of indirect influence, and its influence on emotional and behavioral problems. Based on attachment theory, emotional security theory (EST), and fast history (FH) theory, this study indicated two indirect pathways between destructive IPC and emotional and behavioral problems among Chinese adolescents. The study indicated two mechanisms of influence between destructive IPC and emotional and behavioral problems among Chinese adolescents. Our results showed that destructive IPC does affect emotional and behavioral problems among Chinese adolescents through parent–child attachment and emotional insecurity.

Derived from the overall impact of destructive IPC on Chinese adolescents’ emotional and behavioral problems, the results showed that there is not a significant direct path effect is not between IPC and Chinese adolescents’ emotion–behaviors. In other words, parent–child attachment mediated the mechanism of destructive IPC on Chinese adolescents’ emotional and behavioral problems. Because of the frequent destructive IPC, the interparental relationship grows more tense, which reduces parents’ attention and participation with adolescents. By reducing the level of parent–child attachment, the risk of adolescents’ emotional and behavioral problems is shown to have increased. Therefore, in this study, the direct influence of IPC on Chinese adolescents’ emotional and behavioral problems is not significant, because the influence pathway of IPC and adolescents’ emotional and behavioral problems is mediated by parent–child attachment and emotional insecurity.

This result shows that among Chinese adolescents from primary school and junior high school, the influence of IPC on their emotional and behavioral problems is both immediate and long-terms. Adolescents constantly receive and release negative emotions and even formed aggressive psychology when faced with IPC. Negative emotion, the early stage of aggressive psychology, influences emotional insecurity; however, over time, it will also increase the risk of emotional and behavioral problems affected by the development of parent–child attachment. Therefore, emotional insecurity and parent–child attachment are shown to have played a mediating role in this effecting mechanism. Thus, research hypothesis 1 is not supported, while the other two are supported.

### An indirect pathway through parent–child attachment

Our data showed that there is an indirect pathway in destructive IPC affecting emotional and behavioral problems among Chinese adolescents. In other words, destructive IPC is shown to be positively correlated with emotional and behavioral problems among Chinese adolescents by negatively predicting parent–child attachment. This indirect path has a long-term impact on adolescents. Destructive IPC, as an unfavorable family environment factor, will have a severely destructive impact on parent–child attachment by reducing adolescents’ emotional dependence on parents and parents’ participation (Adare et al., 2021). Secure parent–child attachment can buffer the negative impact of family conflict on adolescents.

This result shows that a series of negative psychological trends brought about by IPC will affect the emotional connection between parents and adolescents, leading to a situation where both do not feel warmth and support each other at home. This will then lead to peer problems or a series of misconduct problems in the process of interpersonal communication. Moreover, frequent interparental conflict increases adolescents’ aggressive behavior and conflict toward their parents. They become irascible, obtain low emotional adjustment ability, and turn incapable to reasonably solve various problems in interpersonal communication. This can then predict the quality of peer relationships and friendships in adolescence (Gasser-Haas et al., 2021), and academic achievement in late childhood and early adolescence. Undoubtedly, this path that influences adolescents’ emotional and behavioral problems through parent–child attachment also tends to be durable and stable over a period of time.

Based on attachment theory, safe attachment relationships formed in early childhood have a huge impact on interpersonal communication, emotional stability, and personal development during adolescence and adulthood. Growing up with favorable rather than unfavorable parent–child attachment enables adolescents to cope better and reduce psychological pressure when faced with adverse life events (Bannink et al., 2013). A study has shown that secure attachment relationship is related to emotional regulation. Adolescents who grow up in a secure and good attachment relationships with either of their parent show better emotional regulation and peer relationship, capable to handle themselves better in peer groups, and indulge in less conduct-related problems (Vu et al., 2016). In a study based on resting-state functional magnetic resonance imaging, it has been emphasized that adolescents with secure parent–child attachment build stronger connections in the limbic system, especially the hippocampus and other nerve tissues, since the hippocampus plays an important role in learning, memory, and emotion. A cross-cultural study pointed out that compared with the United States and Poland, parent–child attachment, especially mother attachment, plays a core role in adolescents’ self-control and social adaptation, and is also considered to have played a greater role in adolescents’ mental health in China and Spain (Mancinelli et al., 2021). Adolescents with secure parent–child attachment have fewer emotional and behavioral problems than those with insecure parent–child attachment. Based on this, our study concluded that parent–child attachment does play a necessary mediating role between destructive IPC and emotional and behavioral problems among Chinese adolescents. Furthermore, based on attachment theory, the emotional distress caused by the perceiving family conflict will spread to the attachment relationship between parents and adolescents. Moreover, it triggers other emotional and behavioral problems in the developing stage of adolescents. Consistent with this specific study’s hypothesis, our study has found an indirect pathway through which destructive IPC is linked to emotional and behavioral problems among Chinese adolescents by influencing parent–child attachment.

### An indirect pathway through emotional insecurity

Our study’s results have found another indirect pathway that states that destructive IPC has a significant positive correlation with adolescents’ emotional and behavioral problems by positively predicting emotional insecurity. This study supports our hypothesis that emotional insecurity does play a mediating role in the relationship between destructive IPC and emotional and behavioral problems.

Emotional security theory (EST) indicated that adolescents who are faced with frequent conflicts in the family have more negative emotional responses (e.g., avoidance and depression) and stress behavior responses (e.g., peer interaction abnormalities, poor academic performance). Stress-induced emotional health and emotional regulation ability are the core parameters of emotional security. Adolescents who are insecure in emotional situations tend to be readily impulsive when dealing with external things that result in emotional and behavioral problems. Adolescents who have witnessed excessive IPC are faced with the risk of structural fragmentation, and collapse of the family system, which produces emotional insecurity, which, in turn, leads to emotional problems, such as sensitivity, depression, avoidance, and failure in forming healthy peer relationships, as well as reduced prosocial behavior and a series of misconduct problems. High stress from destructive IPC can predict negative emotions (Cummings et al., 2006). A study found that the influence of early experiences will be constantly replaced by recent development experience. In other words, it means that in longitudinal studies, as time goes by, the influence of conflict between parents on adolescents’ anxiety level gradually decreases and eventually disappears (Van Eldik et al., 2020)—since adolescents are more susceptible to it than adolescents. Therefore, during the stage of adolescents’ development, those who have witnessed their parents’ conflict are more affected by anxiety than teenagers and adults. This study also provides more support for EST by concluding that emotional insecurity plays a very important role between destructive IPC and Chinese adolescents’ emotional and behavioral problems. Consistent with our research hypothesis, this study has described an indirect pathway through which destructive IPC is linked to adolescents’ emotional and behavioral problems by affecting emotional insecurity.

### Research limitations and prospects

Nonetheless, this study has certain limitations. First, the participants belong to high-grade primary and junior high schools, who are still passing through the development stage in terms of personality, emotion, and mental health level. Although this study is innovative in focusing on the indirect pathways (parent–child attachment and emotional insecurity) of IPC on Chinese adolescents’ emotional and behavioral problems, considering the limitations of cross-sectional studies, the perspective of longitudinal research can allow further opportunities to study the impact of behaviors on adolescents’ future growth, and understand how individual differences and developmental dissimilarity are developed in the healing of conflicts experienced during childhood. Therefore, as an extension to this study, a longitudinal study could be considered to explore other indirect pathways of destructive IPC on emotional and behavioral problems during the child’s adolescence and adulthood. Since this study was conducted in China where citizens tend to pay more attention to the concept of family, the impact of destructive IPC on family structure and its stability, and more importantly, how it manifests among young adolescents, would be greater and more obvious than it is in the West. Therefore, in this study, it is imperative to consider the cultural differences between China and the West.

In addition, during childhood, the factors affecting adolescents’ emotional and behavioral problems are diverse. Under the condition of high-level family instability, parents with low education level will show a higher level of family insecurity (Coe et al., 2017). Therefore, for future research, we aim to more comprehensively consider factors that include family economic level, characteristics of dependents, and so on. An existing study has classified the trajectories of IPC in early childhood into three types: low stability, high-decreasing, and high-increasing marital conflict (Madigan et al., 2016). Therefore, in the future, we would like to classify the trajectory of IPC during childhood, and explore their mechanism of action on adolescents’ emotional and behavioral problems. Some researchers have also indicated that physiological structures are not yet completely mature during infancy and childhood. Exposure to adverse experiences, including destructive IPC and intimate partner violence, will have an impact on adolescents’ brain structure development, such as the Hypothalamus-Pituitary–Adrenal (HPA) axis (Mueller and Tronick, 2019). The relationship between experiencing intimate partner violence (IPV) and adolescents’ emotional and behavioral problems will become stronger over time (Vu et al., 2016). In other words, adverse events in the family environment will have a “sleeper effect” on adolescents’ development. Moreover, destructive IPC will be related to the problematic parenting practices at a higher level each year. Therefore, we aim to focus on the intertemporal impact of destructive IPC on adolescents in the longitudinal study.

Despite these limitations, this study has significantly contributed to the academic study of the influence of IPC on adolescents’ emotional and behavioral problems against the backdrop of Chinese culture. To this end, it opens up two indirect pathways between destructive IPC and adolescents’ emotional and behavioral problems; lays a foundation for more detailed and in-depth research to be conducted in the future; and has significance for Chinese parents to deal with family conflict, stabilize family relations, and cultivate a positive atmosphere for adolescents. In the family environment, it is recognized that conflict between parents will have a huge impact on the mental health and external development of young adolescents. Those growing up in safe, healthy, and happy families will perform well in interpersonal communication, emotional control, psychological resilience, and academic performance.

Since conflicts cannot be avoided in family environments in the future, it is important to apply appropriate and effective solutions during adolescents’ younger years. This indicated that establishment of a stable parent–child relationship and keeping secure emotion can appropriately reduce the impact and pressure caused by destructive IPC. Coping with and changing frequent IPC are the most important aspects in a family environment. Therefore, the important role of adjustment between husband and wife should be considered as a solution to destructive IPC, which reduces its impact on adolescents. The results of our study stress the necessity for continuous and deep concern regarding the link between the family system and Chinese children’s developmental outcomes.

## Conclusion

First, destructive IPC can adversely affect emotional and behavioral problems among adolescents, experiencing more destructive IPC rise the risk of emotional and behavioral problems. Second, destructive IPC plays a damaging role in their emotional security and parent–child attachment, consequently effecting emotional and behavioral problems, Therefore, those Chinese adolescents who experience destructive IPC will experience lower emotional security and parent–child attachment quality, resulting in more emotional and behavioral problems.

## Data availability statement

The original contributions presented in the study are included in the article/supplementary material, further inquiries can be directed to the corresponding author.

## Ethics statement

The studies involving human participants were reviewed and approved by the Ethics Committee of North China University of Science and Technology (Approval No. 2021068). Written informed consent to participate in this study was provided by the participants' legal guardian/next of kin.

## Author contributions

All authors listed have made a substantial, direct, and intellectual contribution to the work and approved it for publication.

## Conflict of interest

The authors declare that the research was conducted in the absence of any commercial or financial relationships that could be construed as a potential conflict of interest.

## Publisher’s note

All claims expressed in this article are solely those of the authors and do not necessarily represent those of their affiliated organizations, or those of the publisher, the editors and the reviewers. Any product that may be evaluated in this article, or claim that may be made by its manufacturer, is not guaranteed or endorsed by the publisher.

## Footnotes

^1^ http://www.sdqinfo.com
